# ABCG2 downregulation in glioma stem cells enhances the therapeutic efficacy of demethoxycurcumin

**DOI:** 10.18632/oncotarget.18018

**Published:** 2017-05-19

**Authors:** Long Chen, Lei Shi, Wenhua Wang, Youxin Zhou

**Affiliations:** ^1^ Department of Neurosurgery, The First Affiliated Hospital of Soochow University, Suzhou 215006, P. R. China; ^2^ Department of Neurosurgery, Traditional Chinese Medicine Hospital of Kunshan, Affiliated Nanjing University of Traditional Chinese Medicine, Suzhou 215300, P. R. China; ^3^ Department of Neurosurgery, The First People's Hospital of Kunshan Affiliated with Jiangsu University, Suzhou 215300, P. R. China

**Keywords:** glioma stem cells, demethoxycurcumin, reactive oxygen species, caspase-3, ABCG2

## Abstract

We analyzed the role of ABCG2, a drug transporter, in determining the sensitivity of glioma stem cells (GSCs) to demethoxycurcumin (DMC). We first demonstrated that ABCG2 is more highly expressed in GSCs than primary astrocytes. Modulation of ABCG2 levels in GSCs by transfection of ABCG2 shRNA or a lentiviral vector encoding ABCG2 revealed an inverse relation between ABCG2 levels and DMC-induced GSC growth inhibition. Suppressing ABCG2 increased DMC-induced apoptosis and G0/G1 cell cycle arrest in GSCs. It also increased levels reactive oxygen species (ROS) in GSCs treated with DMC, resulting in increased cytochrome C and caspase-3 activity. When GSCs transfected with ABCG2 shRNA or overexpressing ABCG2 were xenografted and the tumor-bearing, immunodeficient mice were treated with DMC, ABCG2 expression suppressed the tumor proliferation rate (T/C %). These findings demonstrate that ABCG2 expression is critical for DMC resistance in GSCs and is a potential therapeutic target for GBM.

## INTRODUCTION

Glioma stem cells (GSCs) are responsible for the recurrence of glioblastoma multiforme (GBM) and insensitivity to Temozolomide (TMZ), the first-line drug for GBM treatment. GBM is an aggressive type of malignant glioma with very low 2- and 5-year survival outcomes [1]. There are no effective agents to treat GSCs and the efficacy of adjuvant therapy is limited. TMZ is a 3-methyl derivative of mitozolomide that forms O^6^-methylguanine, which inhibits GBM cell proliferation and induces apoptosis and autophagy in glioma cells [2]. However, TMZ is ineffective in inhibiting GSC proliferation and inducing their apoptosis [3]. Fueyo *et al*. suggested that O^6^-methyl guanine -DNA methyl transferase (MGMT) was responsible for resistance of glioma cells to TMZ and therefore MGMT expression formed the basis for clinical treatment strategies. However, recent studies showed that drug resistance in GSCs was related to ABCG2 and not MGMT [4]. This suggested that the mechanisms of drug resistance in glioma cells and GSCs were different.

ATP-binding cassette sub-family G member 2 (ABCG2) is a xenobiotic transporter that confers resistance to a variety of anticancer drugs by transporting intracellular drugs out of tumor cells. Robey *et al* found that overexpression of ABCG2 lowered intracellular levels of photosensitizers below the threshold required to induce significant tumor cell death [5]. Inhibition of the ABCG2 transporter improved the efficacy of photodynamic therapy on keratinocytes [6]. Recent studies showed that ABCG2 expression was partly responsible for increased resistance of GSCs to chemotherapy. Jia *et al*. reported that inhibition of ABCG2 sensitized CD90^+^ CD133^+^ liver CSCs to chemotherapeutic agents [7]. ABCG2 was also highly expressed in breast cancer stem cells (BCSCs) and its inhibition by 5-aza-2′-deoxycytidine (DAC) sensitized BCSCs to doxorubicin, verapamil, and tamoxifen [8]. Also, cervical cancer stem cells (CSCs), which are characterized by prolonged cell survival, infinite cell proliferation and highly resistant apoptosis became sensitized by silencing ABCG2 expression [9]. Recently, ABCG2 overexpression was confirmed in all GSCs [10, 11]. Xu *et al* reported that high expression of ABCG2 in GSCs reduced accumulation of chemotherapeutic agents and resulted in drug resistance [11]. Also, inhibition of ABCG2 improved the efficacy of sonodynamic therapy (SDT) in GSCs [11]. Jin *et al* reported that high ABCG2 expression in CD133^+^ GSCs conferred mitoxantone resistance [12].

Demethoxycurcumin (DMC) is a major component of *Curcuma longa L*, which effectively inhibited proliferation and induced apoptosis in GSCs *in vitro* [13]. However, its mechanism of action is not fully understood. Therefore, in the current study, we investigated the role of ABCG2 in the chemoresistance of GSCs to DMC and if its downregulation improved therapeutic efficacy of DMC in a mouse xenograft model.

## RESULTS

### ABCG2 expression in primary astrocytes and GSCs

**Figure 1:**
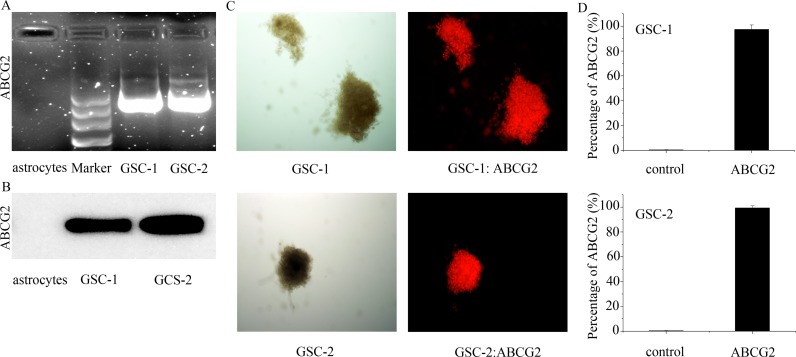
The expression of ABCG2 in the primary astrocytes and GSCs **(A, B)** ABCG2 mRNA and protein levels in primary GSCs as detected by RT-PCR and Western blot, respectively. **(C)** Immunohistochemical analysis showing ABCG2 expression in GSC spheres. **(D)** Flow cytometry analysis of ABCG2 expression in GSC spheres.

Previous research showed that 40-50% WHO III and WHO IV glioma tissues and 100% U251 GSCs overexpressed ABCG2 [11, 12]. Hence, we analyzed ABCG2 expression in primary astrocytes and GSCs by RT-PCR and western blotting. As shown in Figure 1A and 1B, we observed high mRNA and protein expression of ABCG2 in the primary GSCs and no expression in the primary astrocytes. Further, immunohistochemical staining of GSC spheres (Figure 1C) and flow cytometry analysis showed that more than 97% GSC sphere cells were ABCG2-positive (Figure 1B). These results demonstrated that ABCG2 was highly expressed in the GSCs and probably played an important role in their function.

### Association between ABCG2 expression and efficiency of DMC inhibition of GSCs *in vitro*

**Figure 2:**
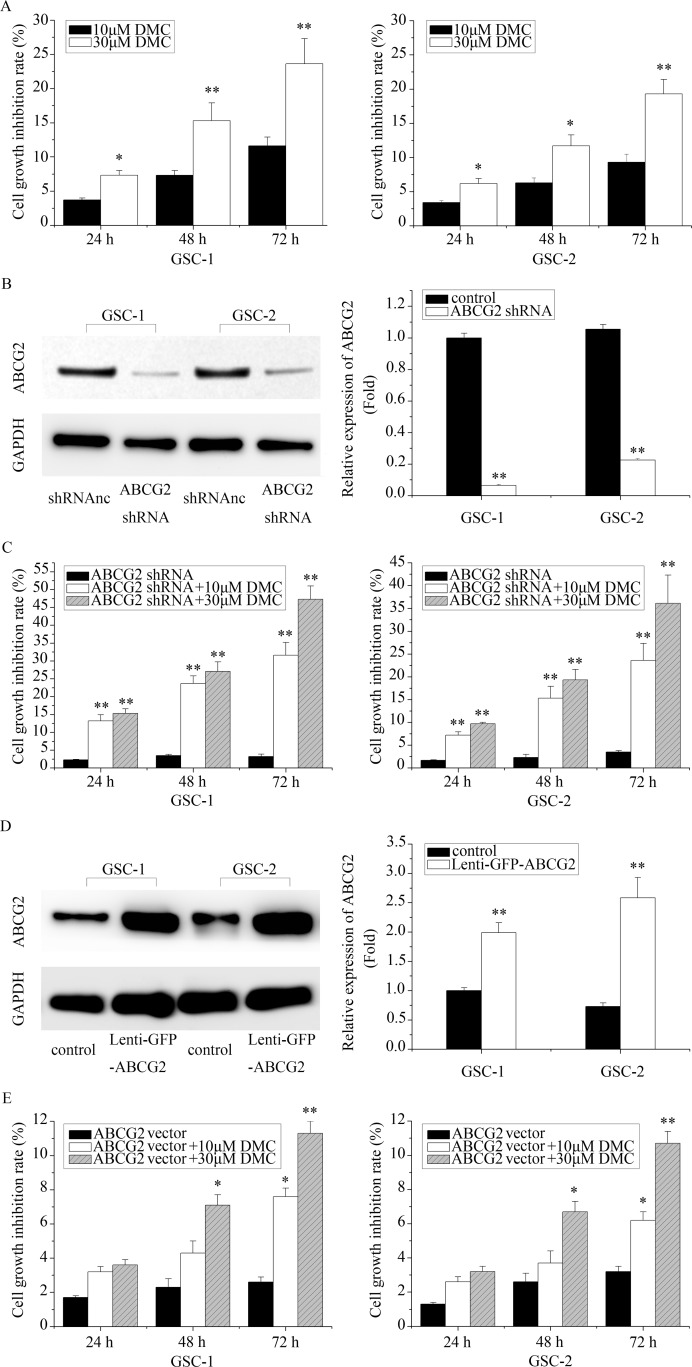
The *in vitro* effects of differential ABCG2 expression on DMC inhibition of GSCs **(A)** The cell growth inhibitory effects of 10μM or 30μM DMC on GSCs as measured by MTT assay. **(B)** Western blot analysis of ABCG2 expression in GSCs transfected with ABCG2 shRNA lentiviral vector. **(C)** The cell growth inhibition rate of 10μM or 30μM DMC on ABCG2 knockdown GSCs (ABCG2 shRNA) as determined by MTT assay. **(D)** Western blot analysis of ABCG2 expression in GSCs transfected with ABCG2 overexpression lentiviral vector. **(E)** The cell growth inhibition rate of 10μM or 30μM DMC on ABCG2 overexpressed GSCs as determined by MTT assay. “Lenti-GFP-ABCG2” is denoted as “ABCG vector”.“Lenti-GFP-ABCG2 shRNA” is denoted as “ABCG2 shRNA”.

Next, we determined the effects of DMC on cell viability of GSCs by MTT assay. As shown in Figure 2A, treatment with 10μM DMC resulted in cell growth inhibition of GSCs by 3.7%, 7.3% and 11.6% at 24, 48and 72 h, respectively. Similarly, 30μM DMC inhibited cell growth of GSCs by 7.3%, 15.3%, and 23.6% at 24, 48 and 72h, respectively.

Further, we investigated if ABCG2 expression influenced DMC-induced GSC growth inhibition. Towards this, we transfected GSCs with lenti-GFP-ABCG2 shRNA and determined that ABCG2 was significantly downregulated in GSCs (Figure 2B). Then, we tested the inhibitory efficiency of DMC in ABCG2 knockdown GSCs. As shown in Figure 2C, treatment of ABCG2 knockdown GSCs with 10μM DMC showed growth inhibition of 13.2%, 23.7% and 31.6% for GSC-1 and 7.2%, 15.3%, and 23.6% at for GSC-2 at 24, 48 and 72h, respectively. When treated with 30μM DMC, the ABCG2 knockdowns GSC1 and GSC-2 showed a growth inhibition rate of 15.3%, 27.1%, and 47.3% and 9.7%, 19.3% and 36.1% at 24, 48, 72 h, respectively. Conversely, we transfected GSCs with ABCG2 overexpressed vector (lenti-GFP-ABCG2) and tested the growth inhibition effects of 10 or 30μM DMC in GSC-1 and GSC-2. As shown in Figure 2D, we observed increased resistance to DMC in ABCG2 overexpressed GSC-1 and GSC-2 compared to the controls. Collectively, these data suggested that ABCG2 expression levels inversely correlated with DMC efficacy in inhibiting GSCs.

### Evaluation of ABCG2 expression on the anti-GSC effects of DMC *in vivo*

**Figure 3:**
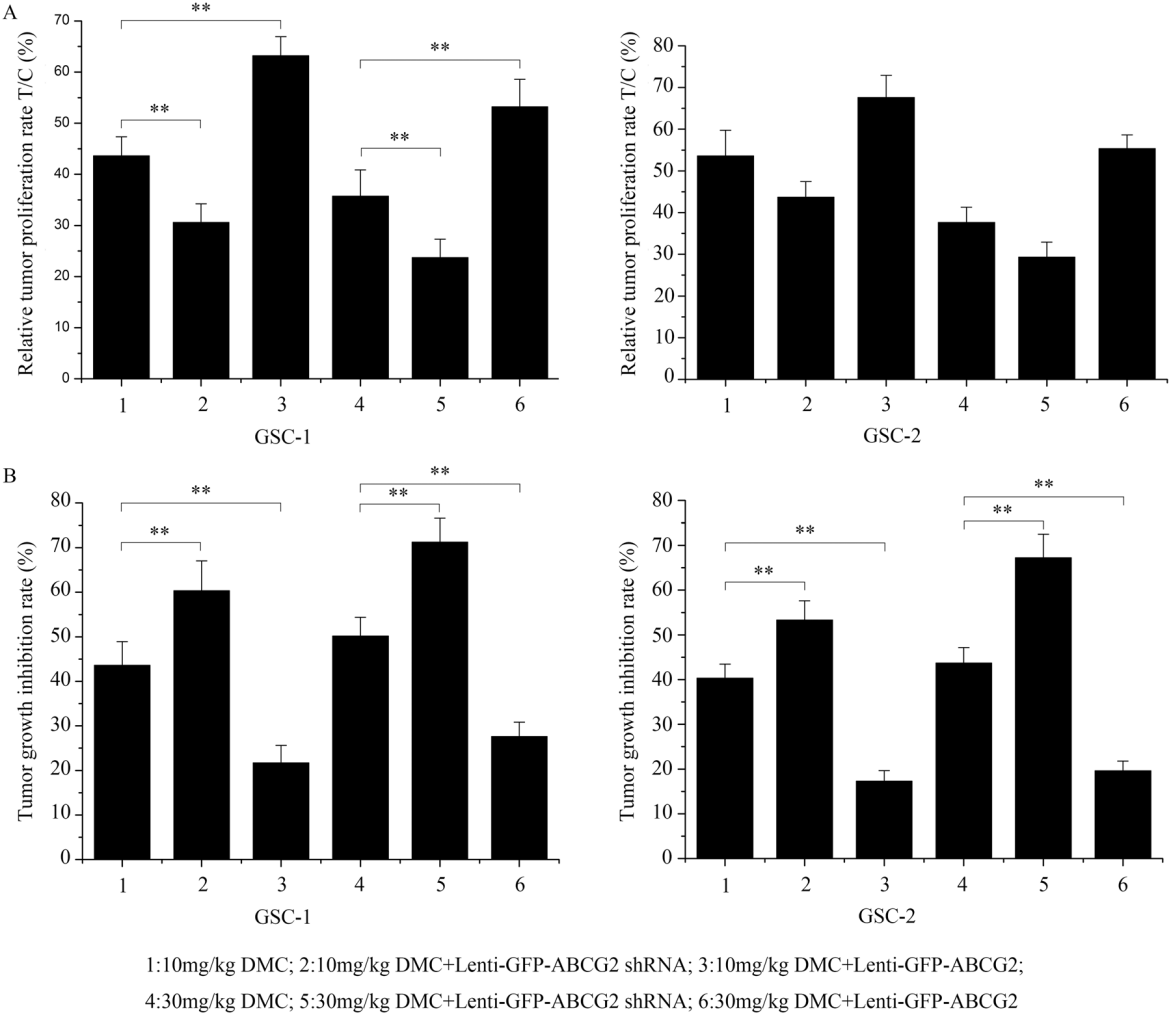
*In vivo* effects of DMC on GSCs with low or high ABCG2 expression Balb/c nude mice were xenografted with GSCs that were transfected with either lenti-GFP-ABCG2 shRNA or lenti-GFP-ABCG2 to downregulate or overexpress ABCG2 in GSCs, respectively. After 30 days, the mice were administered 10mg/kg or 30mg/kg DMC for 30 days. Then, relative tumor proliferation rates T/C % **(A)** and the tumor growth inhibition rates (TGI %) **(B)** were calculated.

Next, we evaluated the *in vivo* relevance of high or low ABCG2 expression on the DMC inhibition of GSCs by implanting 10^6^ CD133-positive GSCs transfected with either ABCG2 shRNA or overexpression lentiviral vectors into immune-deficient nude mice. When the tumor volume reached about 50 mm^3^, the xenograft tumor-bearing nude mice were administered with either 10mg/kg or 30mg/kg DMC. After 30 days, the relative tumor proliferation rate T/C (%) was determined to evaluate the antitumor activity of DMC as described in the methods. As shown in Figure 3A, T/C (%) in 10mg/kg or 30mg/kg DMC-alone treatment group was 43.61% and 35.72% for GSC-1 and 53.61% and 37.62% for GSC-2, respectively. The T/C (%) for ABCG2 knockdown (lenti-GFP-ABCG2 shRNA) GSCs was 30.61% and 23.71% for GSC-1 and 43.71% and 29.31% for GSC-2, respectively for the 10mg/kg or 30mg/kg DMC groups. These data suggested that downregulation of ABCG2 enhanced the anti-tumor activity of DMC on the GSC xenograft tumors. Conversely, the T/C (%) for ABCG2 overexpression (lenti-GFP-ABCG2) GSCs was 63.21% and 53.21% in GSC-1 and 67.62% and 55.36% in GSC-2, respectively for the 10mg/kg or 30mg/kg DMC groups. This suggested that ABCG2 overxpression decreased the anti-tumor activity of DMC on GSCs xenograft tumors.

Further, tumor growth inhibition rate (%) (TGI%) in 10mg/kg or 30mg/kg DMC-alone treatment group was 43.62% and 50.16% in GSC-1 and 40.32% and 43.71% in GSC-2, respectively, whereas ABCG2 knockdown (Lenti-GFP-ABCG2 shRNA) resulted in the TGI% for the 10mg/kg or 30mg/kg DMC groups was 60.32% and 71.23% in GSC-1 and 53.32% and 67.21% in GSC-2, respectively. In contrast, ABCG2 overxpression (Lenti-GFP-ABCG2) resulted in the TGI% for the 10mg/kg or 30mg/kg DMC group were 21.70% and 27.63% in GSC-1 and 17.32% and 19.61% in GSC-2, respectively. These data again showed that ABCG2 expression negatively regulated the tumor growth inhibition rate of DMC on GSCs *in vivo*.

### Evaluation of ABCG2 expression on cell cycle and apoptosis of DMC on the anti-GSC effects

**Figure 4:**
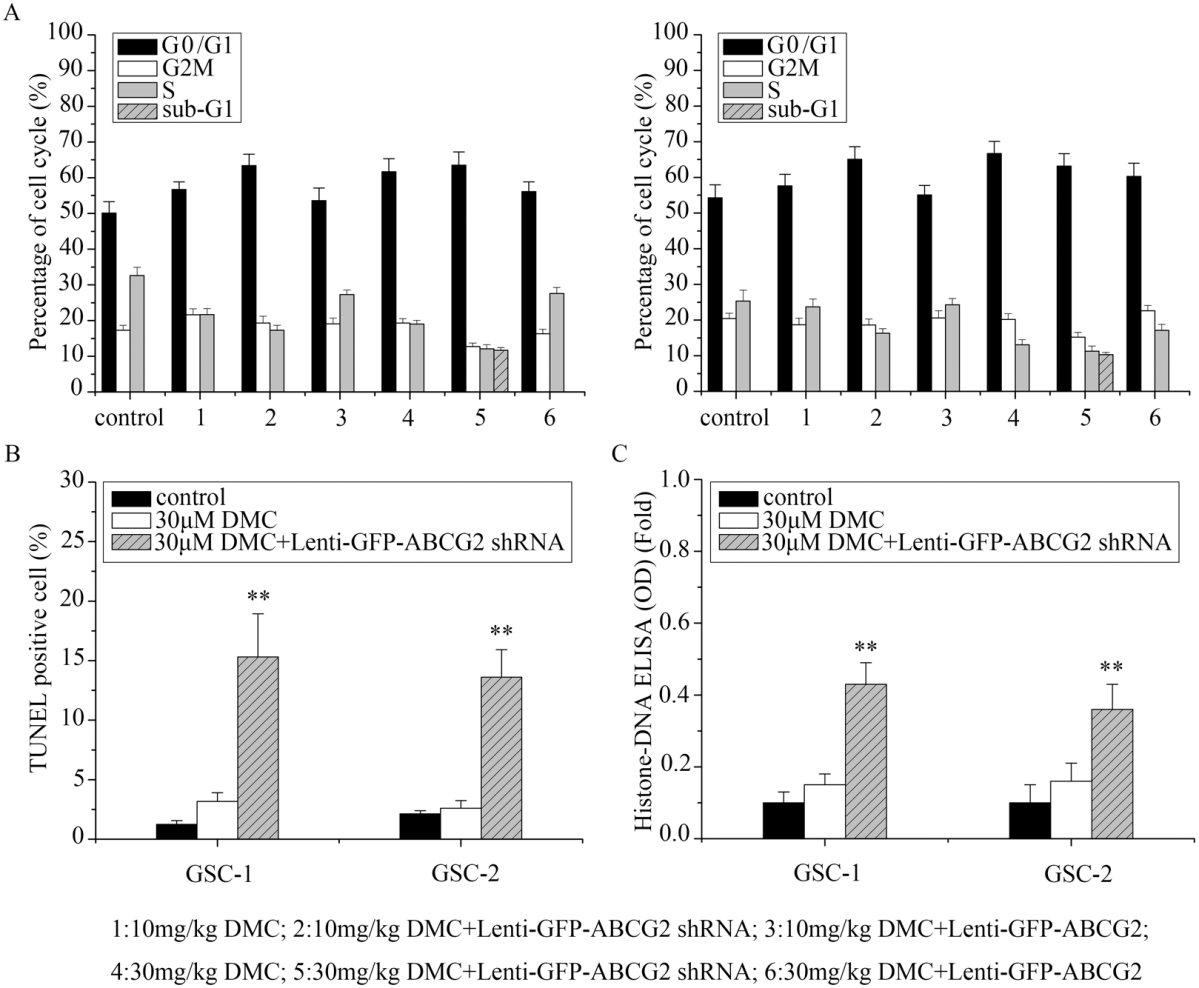
Analysis of cell cycle and apoptosis of GSCs with low or high ABCG2 expression upon DMC treatment **(A)** GSCs were transfected with either lenti-GFP-ABCG2 shRNA or lenti-GFP-ABCG2 to downregulate or overexpress ABCG2 in GSCs and treated with either 10μM or 30μM DMC for 24h followed by PI staining and flow cytometry analysis of the cell cycle. **(B)** TUNEL staining to determine apoptosis of GSCs transfected with or without lenti-GFP-ABCG2 shRNA and treated with 30μM DMC. **(C)** Histone-DNA ELISA analysis of apoptosis in GSCs tranfected with or without lenti-GFP-ABCG2 shRNA and treated with 30μM DMC.

Next, we used flow cytometry to determine if altering ABCG2 levels in GSCs resulted in cell cycle changes upon DMC treatment. Treatment of GSCs to 10μM and 30μM DMC for 48h increased G0/G1 cells proportionately, compared to the controls (P<0.05; Figure 4A). Moreover, ABCG2 downregulation (lenti-GFP-ABCG2 shRNA) further increased G0/G1 phase cells and ABCG2 overexpression (lenti-GFP-ABCG2) lowered G0/G1 phase cells upon treatment of GSCs by 10μM and 30μM DMC. These data suggested that ABCG2 expression levels influenced DMC's ability to inhibit cell cycle in the GSCs.

In Figure 4A, we also observed increased sub-G1 upon 30μM DMC treatment of ABCG2 downregulated (lenti-GFP-ABCG2 shRNA) GSCs, suggesting enhanced apoptosis. To confirm enhanced apoptosis upon ABCG2 downregulation, we treated ABCG2 downregulated GSCs with 30μM DMC and analyzed apoptosis by TUNEL and Histone-DNA ELISA assays. We found that co-administration of lenti-GFP-ABCG2 shRNA and 30μM DMC for 48h increased cell apoptosis of 12.15% in GSCs-1 and 11% in GSCs-2 based on TUNEL assays (Figure 4B), and 0.28-fold in GSCs-1 and 0.2-fold in GSCs-2 based on Histone-DNA ELISA assays (Figure 4C) compared with 30μM DMC treatment. These results suggested that downregulation of ABCG2 enhanced DMC induced apoptosis in GSCs.

### Effects of ABCG2 deficiency on enhancing DMC-induced anti-GSCs was mediated by ROS production and caspase-3 signaling cascade activation

**Figure 5:**
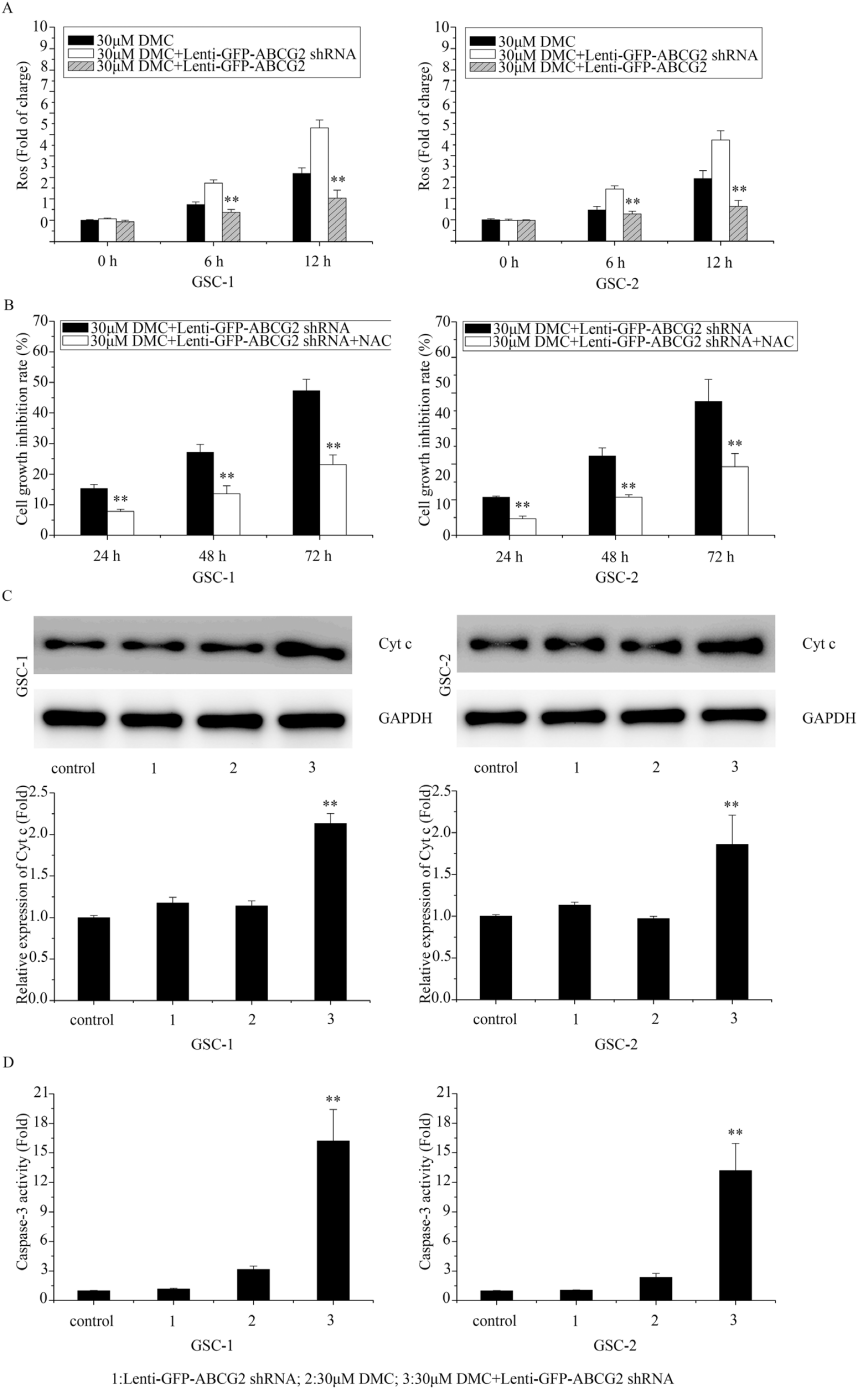
Effects of ABCG2 knockdown on ROS and apoptotic mechanisms upon DMC treatment **(A)** FACS analysis of ROS levels determined in GSCs transfected with or without lenti-GFP-ABCG2 shRNA and treated with 30μM DMC. **(B)** MTT assay analyzing cell growth inhibition rate in GSCs transfected with lenti-GFP-ABCG2 shRNA and treated with 30μM DMC in presence or absence of 10mM NAC. **(C)** Western blot analysis of Cyt c in GSCs transfected with or without lenti-GFP-ABCG2 shRNA and treated with 30μM DMC. **(D)** Caspase-3 activity assay in GSCs transfected with or without lenti-GFP-ABCG2 shRNA and treated with 30μM DMC.

Recently, Shen *et al*. reported that ABCG2 protected against oxidative stress by decreasing ROS generation and enhancing antioxidant capacity [14]. Also, Oliva *et al* showed that low ROS production and tighter mitochondrial coupling influence chemo-resistance to TMZ in glioma [15]. Further, we previously showed that DMC inhibited ROS production [13]. Thus, we postulated that the higher ROS production would increase the inhibitory activity of DMC in ABCG2 downregulated GSCs. As shown in Figure 5A, 30μM DMC treatment enhanced ROS in ABCG2 downregulated (Lenti-GFP-ABCG2 shRNA) GSCs compared to control. In contrast, when GSCs with ABCG2 overexpression were treated with 30μM DMC, ROS levels were lower than the control. These results suggested that overexpression of ABCG2 increased antioxidant capacity of GSCs to DMC whereas downregulation of ABCG2 decreased the antioxidant capacity to DMC.

Further, we treated ABCG2 downregulated GSCs with the antioxidant N-acetyl-L-cysteine (NAC) to block increased ROS induced by 30μM DMC treatment and observed that it resulted in decreased cell growth inhibition rate suggesting that ABCG2 expression modulated ROS levels that were necessary for growth inhibitory effects of DMC on GSCs (Figure 5B).

Since ROS production activates caspase-3 apoptotic signaling cascade [16], we analyzed the cytochrome c (Cyt c) release and caspase-3 activation after 30μM DMC treatment in ABCG2 downregulated GSCs compared to controls. As shown in Figure 5C and 5D, downregulation of ABCG2 enhanced Cyt c levels and caspase-3 activity. In contrast, overxpression of ABCG2 reduced Cyt c (Figure 5C) and caspase-3 activity (Figure 5D) upon DMC treatment. These data suggested that ABCG2 levels influenced DMC induced ROS-dependent activation of caspase-3 mediated apoptosis in GSCs.

## DISCUSSION

In this study, we confirmed that ABCG2 expression in GSCs determined the efficacy of DMC. GSCs are resistant to most chemotherapeutic agents. In glioma cells, drug resistance for TMZ was related to O6- methyl guanine -DNA- methyl transferase (MGMT) expression [17]. However, recent studies have shown that high ABCG2 expression resulted in poor therapeutic effects of TMZ on GSCs [18]. It was noted that the high ABCG2 transporter protein levels increased GSC resistance to chemotherapeutic drugs [19]. ABCG2 pumps various chemotherapeutic drugs out of the cells utilizing ATP hydrolysis and resulted in the drug resistance manifestation of the GSCs [20]. Thus, it was postulated that the ABCG2 levels were related to chemotherapeutic efficacy.

Curcuminoids are yellow and slightly acidic diarylheptanoids that include curcumin and its derivates demethoxycurcumin (DMC) and bisdemethoxycurcumin (BDMC) [21]. All three curcuminoids inhibit tumor cell division and proliferation, with BMDC showing strongest biological activity. However, recent studies have shown that DMC is most effective against gliomas [22]. Many studies have reported that the three monomers of curcuminoids reverse the overexpression of ABC transporters including ABCB1, ABCG2, and ABCC1 in drug resistant tumor cells without causing systemic toxicity [23, 24]. In our previous research, we demonstrated that DMC potently inhibited proliferation and induced apoptosis of GSCs [25]. Thus, we postulated that ABCG2 was involved in drug resistance of GSCs to DMC.

To explore the role of ABCG2 on modulating the chemosensitivity of GSCs, we downregulated or overexpressed ABCG2 in GSCs and tested the effects of DMC *in vitro* and *in vivo*. We observed that ABCG2 was overexpressed in most of CD133^+^ GSCs and its downregulation resulted in increased inhibition of GSC proliferation and enhanced apoptosis by DMC. In contrast, overexpression of ABCG2 in GSCs decreased the sensitivity of GSCs to DMC. Thus, our data demonstrated that ABCG2 represented a more attractive therapy target for GSC chemotherapy.

Low intracellular levels of ROS are critical for normal cell signaling function. However, over production of ROS damages cellular components including proteins, lipids and DNA, which ultimately results in cell death [26]. ROS also play a crucial role in diverse processes of various cancers. Cancer cells often display higher ROS levels that promote cancer cell growth and progression, although it also renders cancer cells more vulnerable to adverse effects of high ROS. In fact, most chemotherapeutic agents kill cancer cells by inducing oxidative stress. Buranrat *et al*. reported that simvastatin and atorvastatin inhibited cancer cell proliferation and induced apoptosis by increasing ROS in KKU-100 cell line of human cholangiocarcinoma [27]. In leukemia cells, docosahexaenoic acid sensitized leukemic lymphocytes to barasertib and everolimus by enhancing ROS and strongly inducing apoptosis [28]. In colon cancer, arctigenin induced apoptosis through increased ROS by activation of the p38 MAPK pathway [29]. Our previous research showed DMC increase ROS in GSCs and triggered a robust increase in GSC apoptosis [25].

However, the molecular pathways involved in DMC-induced ROS that result in cancer cell apoptosis have remained unclear. Recently, Shen *et al*. found that ABCG2 played a protective role against oxidative stress by decreasing ROS generation and enhancing antioxidant capacity in Alzheimer's disease [30]. They showed that ABCG2 overexpression inhibited ROS activation, protected cells from ROS-induced toxicity/death, and the downregulation of ABCG2 lead to increased ROS and inflammatory response. Also, ABCG2 exported cytosolic oxidative molecules and reduced overall cellular oxidative capacity. Thus, we postulated that the selective increase of cellular antioxidant capacity may be one of the possible mechanisms by which ABCG2 protected GSCs against DMC. Our data demonstrated that either upregulation or downregulation of ABCG2 in GSCs resulted in lower or higher levels of ROS, respectively, confirming our hypothesis. Therefore, our study demonstrated that DMC induced oxidative stress in cancer cells and ABCG2 overexpression decreased ROS, thereby reducing the efficacy of DMC in GSCs. We demonstrated that knockdown of ABCG2 restored high ROS levels and therefore enhanced sensitivity of GSCs to DMC. Ding *et al* reported that increased ROS resulted in extensive oxidative DNA damage that upregulated pro-apoptotic Bax and cleaved caspase-3, implying that increased ROS activated caspase-3 signaling [31]. Therefore, we analyzed the expression of Bax, Cyt c and caspase-3 and confirmed that DMC treatment in GSCs with ABCG2 knockdown increased Cyt c levels and caspase-3 activity whereas GSCs with ABCG2 overexpression decreased Cyt c levels and caspase-3 activity during DMC treatment.

In summary, we demonstrated that modulating the expression of ABCG2 altered the efficacy of DMC therapy on GSCs by regulating ROS production and thereby influencing the caspase-3- apoptotic pathway. Hence, ABCG2 is critical for DMC resistance in GSCs and is a potential therapeutic target for GBM.

## MATERIALS AND METHODS

### Primary glioblastoma cell cultures

Human glioblastoma tissues were obtained after informed consent from 2 adult patients diagnosed with WHO grade IV glioma. Primary glioblastoma cell cultures were obtained after mechanical dissociation according to the protocol described by Darling *et al* [32]. The cells were subcultured in DMEM with 2% fetal calf serum (FCS) to prevent the growth of contaminating rodent fibroblasts for 1 week after which the cells were cultured in 10% FCS and antibiotics of Penicillin and Streptomycin. Glial origin was confirmed by morphology and staining with the anti-glial fibrillary acidic protein (GFAP) monoclonal antibody (mAb) clone 6F2 (Dako, Glostrup, Denmark). All experiments on these cells were performed before passage 5.

### Magnetic cell separation of CD133-positive cells

Primary gliostoma cells were dissociated from cell culture dishes and resuspended in PBS with 0.5% BSA and 2mM EDTA and incubated with CD133/1 microbeads (Miltenyi Biotech) followed by positive magnetic cell separation using several MACS columns. The purified CD133^+^ cells were stained with anti- CD133/2-PE (Miltenyi Biotech) and analyzed on a BD FACSCalibur. Then, neurosphere cultures were obtained by growing CD133^+^ tumor cells in stem cell-permissive DMEM/F12 medium supplemented with 20ng/ml each of human recombinant epidermal growth factor and human recombinant basic fibroblast growth factor (R and D Systems), and human leukemia inhibitory factor (Chemicon) and 2% B27 (Life Technologies). These culture conditions enabled tumor cells to retain the molecular characteristics of the primary tumor, with only minor changes in differentiation, expression pattern, and genetic mutation profile.

Cultured primary astrocytes were generated from a slightly injured brain tissue fragment obtained after consent from a patient with cerebral trauma. The grey matter was dissected and dispersed repeatedly after washing in PBS. Primary astrocytes were cultured according to the protocol described by Darling *et al* [32].

### MTT cell growth assay

To assess the effects of TMZ and/or DMC, 5×10^3^ CD133^+^ GSCs/well were plated in 96-well plates with six replicate wells at the indicated concentrations of TMZ and/or DMC for 12, 24, 36, 48 and 72 h, respectively. MTT assay was performed to analyze the cell proliferation as described previously [10].

### FACS analysis of cell cycle

CD133^+^ cells that were treated with TMZ and/or DMC were trypsinized and subsequently fixed with 70% ice-cold ethanol for 1h. After PBS washing, the cells were stained in HBSS containing 50μg/ml Propidium Iodide (PI) (Sigma-Aldrich) and 50μg/ml of RNase A (Boehringer Mannheim, Indianapolis, IN), for 1h at room temperature, and analyzed by FACScan (Becton-Dickinson, San Jose, CA). The cell cycle analysis was performed by ModFit LT software [11, 12].

### Caspase-3 activity assay

The Caspase-3 activity was determined using the Caspase-3 activity kit (Beyotime Institute of Biotechnology, Haimen, China). The cells from various samples were homogenized in 100μl reaction buffer (1% NP40, 20mMTris-HCl (pH 7.5), 137mM NAD and 10% glycerol) containing 10μl 2mM Ac-DEVD-pNA (Caspase-3 substrate) and incubated at 37°C for 2h. Caspase-3 activity was measured with an ELISA reader at an absorbance of 405 nm.

### TUNEL staining for morphological analysis of apoptosis

For TUNEL analysis, cells that were treated with DMC and/or ABCG2 shRNA for 24 and 48h were fixed with 4% paraformaldehyde for 30 min before incubating with *In Situ* Cell Death Detection Kit, Fluorescein (Roche, Palo Alto, CA). Cells were analyzed and enumerated by fluorescence microscopy [13].

### ELISA asssays of cell apoptosis

The Cell Apoptosis ELISA Detection Kit (Roche, Palo Alto, CA) was used to detect Histone-DNA in GSCs after different treatments according to the manufacturer's protocol. Also, the PathScan® Phospho-Stat3 (Tyr705) Sandwich ELISA kit was employed to detect p-STAT3 (Cell Signaling Technology Inc., Danvers, MA) according to the manufacturer's protocol. Briefly, after indicated treatments, cells from different samples were lysed in ice-cold lysis buffer and bound to the immobilized first detection antibody on the ELISA plates. Subsequently, the peroxidase-conjugated secondary antibody was added to each well. After adding the peroxidase substrate, the Histone-DNA and p-STAT3 levels were determined spectrophotmetrically at 405 and 450 nm, respectively.

### ROS production detection

To quantitate ROS in GSCs, cells were loaded with 1μM fluorescent dye dihydrorhodamine for 2h before drug treatments. After the drug treatments, GSCs were suspended in PBS on ice, fixed by 70% Ethanol at -20°C and analyzed by FACS.

### *In vivo* mouse xenograft tumor model

*In vitro* cultured CD133^+^ GBM cells (2-5x 10^6^ in 0.1 ml) were injected subcutaneously into the flanks of 3-5 week old Balb/c nude mice (n=8/group; 18-22g). After the tumor was established (about 50-75 mm^3^), as determined by caliper measurements, the mice were randomly distributed into the following 4 experimental groups: (a) DMSO control: 100μL DMSO/PBS (1:1) ; (b) TMZ-only: 20mg/kg TMZ in 100μL DMSO/PBS; (C) DMC-only: 10 or 30mg/kg DMC in 100μL DMSO/PBS; and (iv) 20mg/kg TMZ plus 10 or 30mg/kg DMC in 100μL DMSO/PBS. The treatments were given daily for 24 days. Then, tumor volumes were calculated using the following formula: tumor volume (TV) = (length × width^2^) × 0.5. Based on the absolute tumor volume measurements, we calculated the relative tumor volume (RTV) as RTV = V_t_/V_0_ (V_0_, the measured tumor volume at day 0; Vt, the measured tumor volume at each time point). Further, the relative tumor proliferation rates (T/C %) were calculated using the following formula: T/C % =(T_RTV_/C_RTV_) x 100%, (T_RTV_: the treatment group RTV; C_RTV_: model control group RTV). Also, the tumor growth inhibition rates (TGI %) were calculated using the following formula: Tumor growth inhibition rate =(Average tumor weight of experimental group-Average tumor weight of model group)/Average tumor weight of model group x 100%. All mice procedures were conducted in accordance with the Animal Care guidelines by the First People's Hospital of Kunshan affiliated with Jiangsu University. After 30 days, tumor samples were snap-frozen for further experiments.

### Western blot analysis

For western analysis, GSC cells and tumor samples were solubilized in the protein lysis buffer (137mM NaCl, 15mM EGTA, 0.1mM sodium orthovanadate, 15mM MgCl2, 0.1% Triton X-100, 25mM MOPS, 100μM phenylmethylsulfonyl fluoride and 20μM leupeptin, adjusted to pH 7.2). Then, 30μg protein per sample was subjected to 12% SDS-PAGE using the Laemmli discontinuous buffer system (Bio-Rad Laboratories, Richmond, CA). The electrophoresed proteins were then transferred to a PVDF membrane and then incubated overnight with anti-human primary antibodies for Cyt c (AC909, Beyotime Biotechnology Inc., Nanjing, China) and ABCG2 (sc-18841, Santa Cruz Biotechnology, Santa Cruz, CA). Then, after 1X TBST washes, the membranes were incubated with the secondary antibodies (A0216, Beyotime Biotechnology Inc., Nanjing, China) for 1h. The blots were developed with enhanced chemiluminescence (Amersham Life Science, Arlington Heights, IL). For control, the membranes were reprobed with a primary GAPDH antibody (sc-47724, Santa Cruz Biotechnology, Santa Cruz, CA) and further developed.

### Statistical analysis

All statistical tests were performed using SPSS Graduate Pack 11.0 software (SPSS, Chicago, IL). The mean and SE, in addition to one-way ANOVAs were determined to analyze significant differences between groups of data. P < 0.05 was considered statistically significant.
